# Green-to-Red Photoconversion of GCaMP

**DOI:** 10.1371/journal.pone.0138127

**Published:** 2015-09-18

**Authors:** Minrong Ai, Holly Mills, Makoto Kanai, Jason Lai, Jingjing Deng, Eric Schreiter, Loren Looger, Thomas Neubert, Greg Suh

**Affiliations:** 1 Department of Cell Biology, Skirball Institute of Biomolecular Medicine, New York University, School of Medicine, New York, New York, United States of America; 2 Department of Biochemistry and Molecular Pharmacology, Skirball Institute of Biomolecular Medicine, New York University, School of Medicine, New York, New York, United States of America; 3 Janelia Research Campus, Howard Hughes Medical Institute, Ashburn, VA, United States of America; AntiCancer Inc., UNITED STATES

## Abstract

Genetically encoded calcium indicators (GECIs) permit imaging intracellular calcium transients. Among GECIs, the GFP-based GCaMPs are the most widely used because of their high sensitivity and rapid response to changes in intracellular calcium concentrations. Here we report that the fluorescence of GCaMPs—including GCaMP3, GCaMP5 and GCaMP6—can be converted from green to red following exposure to blue-green light (450–500 nm). This photoconversion occurs in both insect and mammalian cells and is enhanced in a low oxygen environment. The red fluorescent GCaMPs retained calcium responsiveness, albeit with reduced sensitivity. We identified several amino acid residues in GCaMP important for photoconversion and generated a GCaMP variant with increased photoconversion efficiency in cell culture. This light-induced spectral shift allows the ready labeling of specific, targeted sets of GCaMP-expressing cells for functional imaging in the red channel. Together, these findings indicate the potential for greater utility of existing GCaMP reagents, including transgenic animals.

## Introduction

Fluorescent proteins (FP) have dramatically expanded options for imaging biological samples. Two of the most broadly adopted FP-based technologies are genetically encoded biosensors for real-time monitoring of specific analytes in living specimens [1, 2], and photoactivatable/photoswitchable fluorescent proteins (paFPs) for super-resolution imaging and highlighting/time-lapse imaging [3–6].

The most widely used biosensors are genetically encoded calcium indicators (GECIs) [1, 2, 7, 8]. Among GECIs, those based on the GCaMP scaffold [2], consisting of circularly permuted GFP (cpGFP) fused to calmodulin (CaM) and a Ca^2+^/CaM-binding myosin light chain kinase fragment (M13), are the best calibrated. Recent advances in GCaMP engineering have produced large increases in sensitivity and signal-to-noise ratio [9, 10]. Moreover, GCaMP/GECI variants with different emission spectra have been developed [11, 12].

Photoactivatable/photoswitchable proteins have been both identified in nature and engineered. These proteins, such as Kaede [4] Dendra [13], EosFP [5] and photoactivatable GFP (paGFP) [3] undergo light-induced changes in fluorescence intensity and/or wavelength, and allow target labeling with high spatio-temporal precision [4, 14, 15].

In this study, we found that commonly used GECIs (GCaMP3, GCaMP5G and GCaMP6s) can be readily photoconverted from green to red through exposure to blue-green light. The resulting red GCaMPs remain calcium-sensitive. We also identified amino acid residues important for this photoconversion and generated a GCaMP variant with increased photoconversion efficiency in cell culture. That is, we have shown that high-performance GECIs in common usage can function as “photoactivatable GECIs”, allowing the specification of target cell populations or organelles for functional imaging. Thus, two of the most useful features of fluorescent proteins can be combined in single reagents.

## Results

### Green-to-red photoconversion of GCaMP3

**Fig 1:**
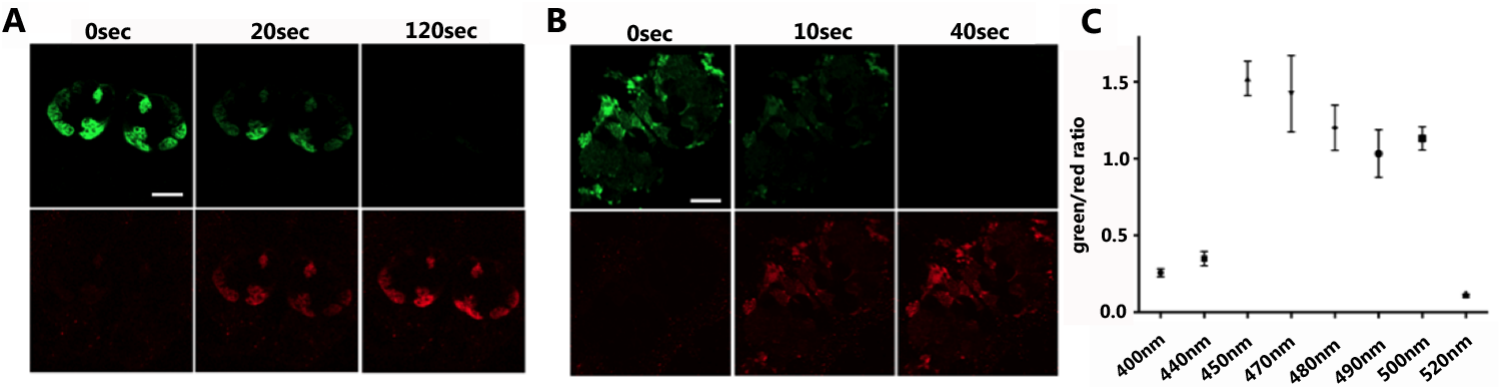
GCaMP3 is converted from green to red fluorescence after exposure to blue light. **(A-B)** Fluorescent micrographs of dissected *Drosophila* brains expressing UAS-GCaMP3; *IR8a*-GAL4 **(A)** or UAS-GCaMP6s; NP225-GAL4 **(B)**. The dissected brains were exposed to blue light (mercury arc light passed through a Zeiss 63x oil-immersion objective) for different durations as indicated at the top of each panel. Green (top) and red (bottom) fluorescence micrographs were captured using a confocal microscope. Scale bar: 20μm. **(C)** Green-to-red photoconversion of GCaMP3 as quantified by the ratio of red-to-green fluorescence intensity (y-axis) following exposure to light from a Xenon lamp (x-axis).

During a routine microscopy experiment, we serendipitously found that the fluorescence of GCaMP3 expressed under control of the *IR8a*-GAL4 driver [16, 17] in *Drosophila* olfactory sensory neurons converted from green to red after ~20 sec exposure to blue-green light from a mercury lamp (Fig 1A). Prolonged light exposure (>2 min) resulted in the complete conversion of green fluorescence to red (Fig 1A). The green-to-red photoconversion of GCaMP3 also occurred in other fly neurons including neurons in the central brain (S1 Fig). We found that improved GCaMP variants, including GCaMP5G [18] and GCaMP6s [10], also underwent green-to-red photoconversion upon exposure to light–as short as 10 seconds (S2B Fig and Fig 1B). After 40 seconds of light exposure, green fluorescence of GCaMP6s was completely photoconverted to red (Fig 1B).

We then investigated the possibility that the photoconversion of GCaMP3 can also occur in mammalian cells. We found that GCaMP3 underwent green-to-red photoconversion in HEK293 cells (S2A Fig), although requiring at least 2 minutes of light exposure.

To identify the wavelengths of light responsible for the green-to-red photoconversion, we used various sets of filters to limit the wavelength of light emitted by the mercury lamp. We found that light passing through a DAPI, TRITC or Cy5 filter did not photoconvert GCaMP3, but light passing through a FITC filter–i.e. blue light—was able to do so. We then used light generated from the xenon lamp to more precisely define the range of photoconverting wavelengths. In doing so, we found that light from 450 nm to 500 nm induced the green-to-red photoconversion of GCaMP3, with roughly uniform efficiency in this range (Fig 1C).

### Calcium responsiveness of the converted red GCaMPs

**Fig 2:**
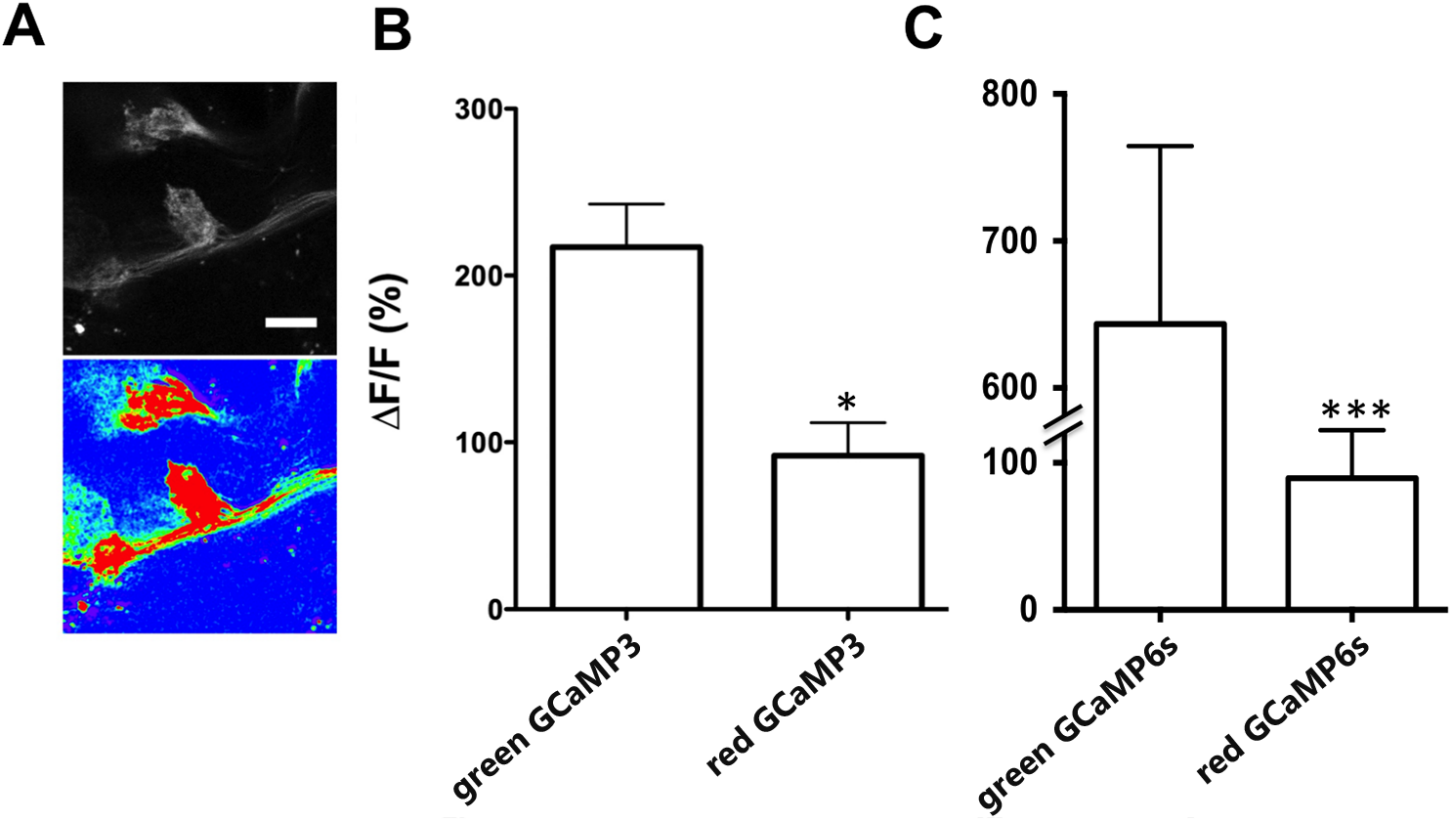
Photoconverted GCaMP3 and GCaMP6s retain calcium sensitivity. Calcium imaging of a dissected fly brain expressing *IR8a*-GAL4 and UAS-GCaMP3 in response to depolarizing reagent, 40mM KCl. Images of red fluorescence from a brain before (top panel) and after (bottom panel, pseudo-colored) KCl stimulation are shown. Scale bar: 10μm. **(B)** Quantification of changes in GCaMP3 fluorescent intensity (ΔF/F) in response to KCl depolarization. **(C)** Fluorescent intensity (ΔF/F) in response to KCl was measured in the brains of flies harboring UAS-GCaMP6s and *NP225*-GAL4, which expresses in central neurons. Student’s *t*-test. Error bars represent SEM. *p<0.05; ***p<0.01. n > 4 for each experimental group.

Having shown that GCaMPs undergo green-to-red photoconversion, we determined whether the photoconverted (red) GCaMP3 species retains Ca^2+-^dependent fluorescence. To this end, we converted a subset of GCaMP3-expressing antennal lobe neurons and imaged both populations in response to activation. Exposure to high potassium (KCl) increased fluorescence intensity of the converted red GCaMP3 by 90±20% compared with 220±25% (ΔF/F; s.e.m.; N = 6) for unconverted GCaMP3 (Fig 2A and 2B). We observed similar results with GCaMP6s expressed in a population of neurons in the central brain (Fig 2C). Our findings indicate that common GCaMPs retain calcium responsiveness when their fluorescence is converted from green to red, albeit with reduced sensitivity.

### Amino acid residues critical for photoconversion

To identify amino acid residues in GCaMP3 required for photoconversion, we tested whether other variants of GCaMPs can be photoconverted from green to red. We found that GCaMP1.6 [19] expressed in fly neurons and GCaMP2 [20] expressed in HEK293 cells could not be photoconverted from green to red by exposure to light (S2A and S2B Fig). By contrast, GCaMP5G [18] (S2A Fig) and all variants of GCaMP6 [10] did undergo this photoconversion.

**Fig 3:**
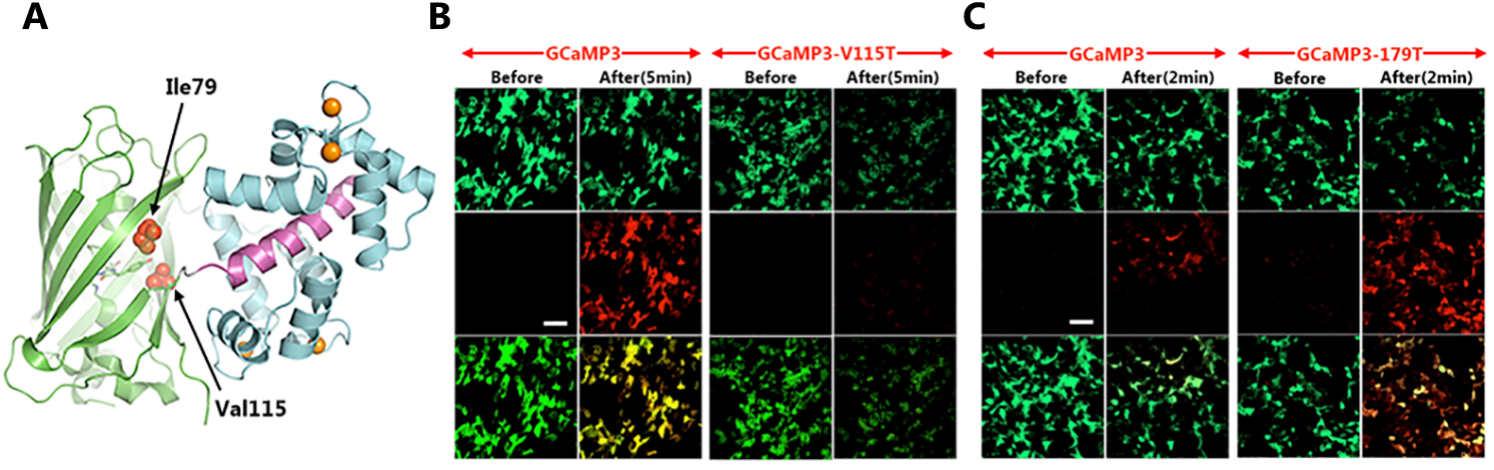
The amino acid residues critical for green-to-red photoconversion. **(A)** A 3-D crystal structure of GCaMP3 protein illustrating GFP backbone in green, calmodulin in cyan and myosin M13 in purple. The spatial positions of amino acid Ile79 and Val115 are highlighted by arrows. **(B-C)** Control and mutant GCaMP3s were expressed in HEK293 cells. Green (top), red (middle) and merged (bottom) fluorescent micrographs were taken before and after the cells were exposed to blue light (mercury arc light passed through a FITC filter) for either 5 minutes **(B)** or 2 minutes **(C)**. Note that V115T results in a loss of green-to-red photoconversion, whereas I79T improves efficiency. Scale bar: 20μm.

We then compared the amino acid sequences in the non-photoconvertible GCaMP2 and the photoconvertible GCaMP3. GCaMP3 has three point mutations near the cpGFP domain: M66K, T116V and N363D. Given that the arginine at position 2 in GCaMP2 was deleted in GCaMP3, these mutated amino acid residues correspond to K65, V115, and D362 in GCaMP3. V115 in GCaMP3 is located close to the chromophore in the protein structure [21, 22] (Fig 3A). Based on this observation, we hypothesized that the T116V mutation was the key to the photoconvertibility of GCaMP3. To test this, we reverted V115 of GCaMP3 back to T115 (as in GCaMP2) and expressed the mutant construct in HEK293 cells. Indeed, the resulting GCaMP3 variant (GCaMP3-V115T) retained green fluorescence, but could not be photoconverted as before (Fig 3B). Similarly, GCaMP3-V115A could not be photoconverted (Table 1). Mutation of V115 to G or W resulted in a complete loss of green fluorescence (Table 1). These results demonstrate that V115 is critical for the photoconversion of green GCaMP3 fluorescence to red.

**Table 1:** Summary of Photoconvertibility of GCaMP3 Variants.

Mutations	Green Fluorescence	Convertible to red
**I79T**	**Yes**	**Yes** *
**I79G**	**No**	**ND**
**V115T**	**Yes**	**No**
**V115A**	**Yes**	**No**
**V115G**	**No**	**ND**
**V115W**	**No**	**ND**
**S117A**	**Yes**	**Yes**
**T222H**	**No**	**ND**
**V225N**	**No**	**ND**

ND: Not done

*Higher conversion efficiency compared to GCaMP3.

To determine whether other amino acid residues located proximal to the chromophore are required for photoconversion, we made additional point mutations (Table 1). Several of these mutations—including I79G, T222H and V225N—resulted in a loss of green fluorescence (Table 1). Notably, one mutation, I79T, resulted in increased efficiency in green-to-red photoconversion with green-fluorescent GCaMP3-I79T readily converting to red within 2 minutes of light exposure in cultured HEK293 cells, compared with >5 minutes required for wild-type GCaMP3 (Fig 3C).

### Anaerobic cellular environments promote GCaMP photoconversion

**Fig 4:**
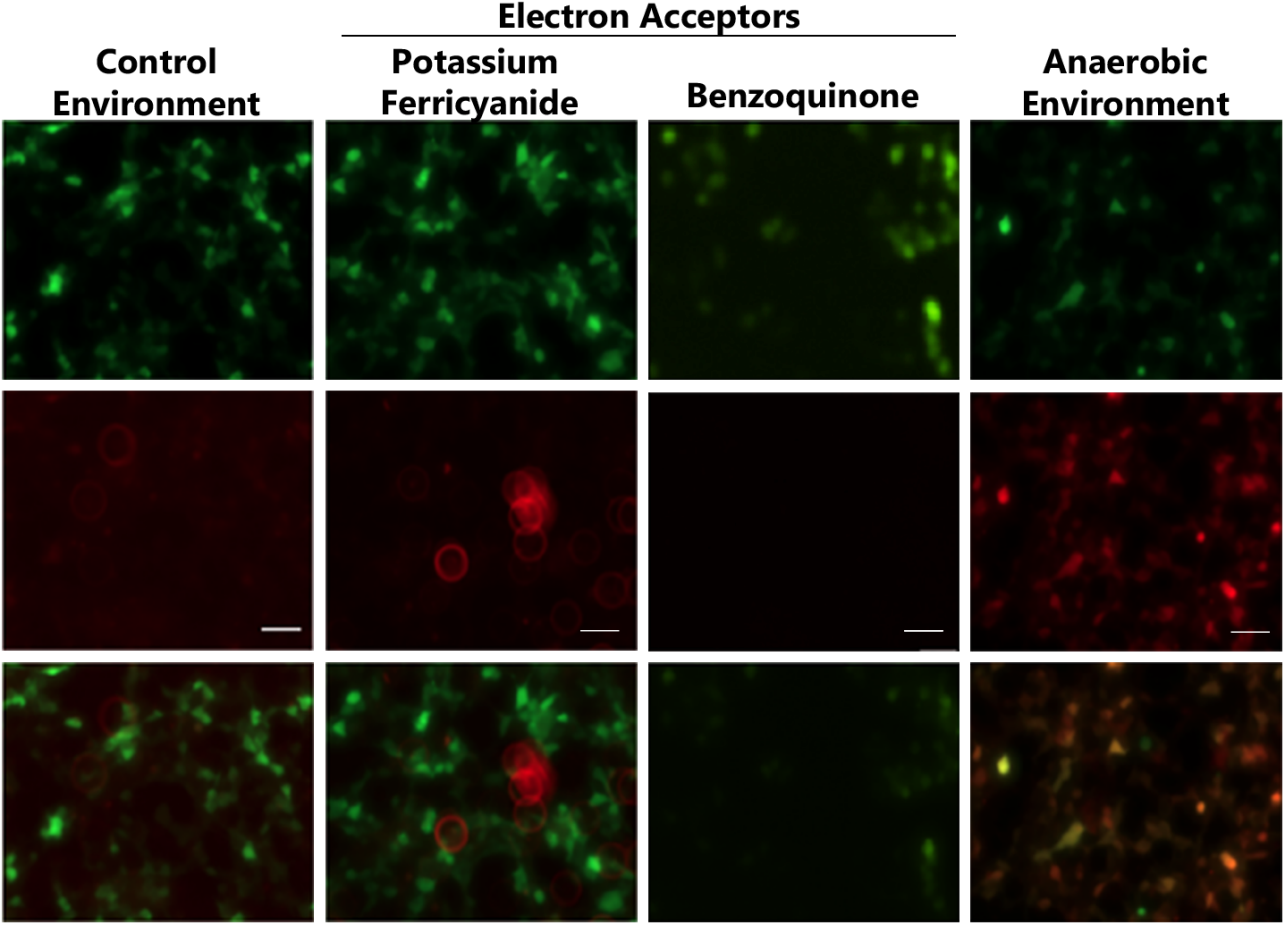
The efficiency of GCaMP3 photoconversion in HEK293 cells is dramatically enhanced under low-oxygen condition. GCaMP3-expressing HEK293 cells were illuminated first under control culture conditions, with the presence of electron acceptor potassium ferricyanide or benzoquinone (both at 5mM), or under low-oxygen conditions created by treating the cells with 30μg/ml catalase, 4.5mg/ml glucose and 250μg/ml glucose oxidase for 30 minutes. Cells were then exposed to blue light for 2 minutes. Green (top), red (middle) and merged (bottom) fluorescent micrographs. Scale bar: 20μm.

A similar light-induced shift of the GFP emission spectrum from green to red has been reported [23–25]. Consistent with this, we found that *Drosophila* central complex neurons expressing membrane-localized mCD8-GFP were indeed converted from green to red with blue light exposure (S3 Fig). Two possible mechanisms have been proposed for the light-induced shift in GFP fluorescence from green to red: “anaerobic redding” [23] and “oxidative redding” [26]. To determine whether either mechanism could account for the green-to-red photoconversion observed for GCaMP, we expressed GCaMP3 in HEK293 cells and measured photoconversion efficiency under various conditions. We found that the green-to-red photoconversion of GCaMP3 occurred more efficiently under low-oxygen conditions produced by the presence of oxygen-depleting reagents (Fig 4). Replenishing photoconverted HEK293 cells with fresh medium lacking these oxygen-scavenging reagents did not result in observable decay in red florescence after one hour (data not shown), suggesting that the converted red GCaMP3 is stable. Additionally, photoconverted HEK293 cells had no obvious morphological abnormalities and no observable difference in cell death compared to controls one hour post-conversion. By contrast, the presence of electron acceptors in the culture medium of HEK293 cells expressing GCaMP3 had no effect on the efficiency of GCaMP photoconversion (Fig 4). These results indicate that an anaerobic environment promotes the green-to-red photoconversion of GCaMPs observed here. By contrast, photoconversion of purified GCaMP protein required electron acceptors such as potassium ferricyanide, and we observed that red fluorescence decayed a few hours after photoconversion (S4 Fig). These results may suggest that GCaMPs inside cells undergo a different mechanism of photoconversion from purified GCaMP protein.

## Discussion

We report here that the fluorescence of the widely used GCaMP calcium sensors can be readily converted from green to red by exposure to blue-green light (450 nm to 500 nm). This photoconversion is irreversible and occurs in both insect and mammalian cells. Most importantly, the resulting red GCaMP remains responsive to changes in [Ca^2+^] levels. We identified amino acid residues in GCaMP critical for photoconversion. We also generated a GCaMP-I79T mutant with higher photoconversion efficiency.

The green-to-red photoconversion of GCaMPs likely involves a mechanism other than that used by other photoconvertible fluorescent proteins such as EosFP [5], Kaede [4, 27], Dendra [28], KikGR [29] and mMaple [30]. Crystal structures of these fluorescent proteins suggest similar mechanisms for green-to-red photoconversion. For example, Kaede undergoes light induced cleavage of the peptide bond at the His residue of the His-Tyr-Gly chromophore. This results in extended electron π-conjugation into the imidazole group of the chromophore His residue, leading to a spectral shift from green-to-red [27].

Unlike naturally occurring photoconvertible fluorescent proteins, however, GCaMP has a chromophore containing the tri-peptide sequence Thr-Tyr-Gly. The mechanisms of light-induced “redding” of GFP and GCaMP are not completely understood but may be elucidated through structural analysis in the future. Given that the photoconverting light (450 nm—500 nm) is of very different wavelength than that used to activate paGFP (i.e 405 nm), the mechanism likely proceeds via a different path than decarboxylation of GFP-Glu222, as occurs with paGFP [31].

Campbell and colleagues generated green-to-red photoconvertible GECIs by fusing circularly permuted mMaple to CaM and M13, which they named GR-GECOs [32]. GR-GECOs can be photoconverted from green-to-red by exposure to violet light (405nm). Both the green and red states of GR-GECOs respond to calcium [32]. Recently, mutants of GCaMP6s and GCaMP6f have been designed that exhibit 405 nm-dependent dark-to-green photoactivation [33]. These “PA-GCaMPs” are based on the paGFP [3–6] mutant and the recognition that reverting the “eGFP” positions increases near-UV absorption. Finally installation of the “superfolder” GFP [34] mutations was required to restore folding and stability.

Employing green-to-red photoconversion of “wild-type” GCaMPs may offer several advantages over the use of GR-GECOs or PA-GCaMPs. First, given that GCaMPs have been widely used and GCaMP-related transgenic animal models are readily available, green-to-red photoconversion of GCaMP can be readily utilized in many labs, without the need to create additional lines. Second, green-to-red photoconversion of GCaMPs is possible with longer wavelength light (450 nm–500 nm) as opposed to 405nm for GR-GECO and PA-GCaMPs, resulting in much greater penetration and decreased phototoxicity.

Green-to-red conversion of GCaMPs could have many applications. First, it could be used for *in vivo* calcium imaging of spatially-specified subsets of neurons (or other cells), such as those projecting to a particular target brain or peripheral organs [35]

Second, this technique could be used to label specific populations of cells (e.g. migratory neurons, stem cells or neuronal precursors) during development and both follow and functionally record from the cells. In addition to neuronal cells, the green-to-red conversion of GCaMPs could be used to visualize tumor-host interactions, proliferating and metastasizing cancer cells surrounded by normal cells [36].

Third, it could be used in cell biology studies; for example, specific subcellular organelles [37] expressing GCaMP could be selected for functional imaging based on spatial restriction of photoconverting light, rather than case-by-case targeting strategies.

Expression of both a compatible GCaMP and a PA-GCaMP followed by spatial separation of 405 and 500 nm converting light could allow specification of both green and red volumes of interest, facilitating 2-color activity imaging of defined subsets. In addition to the potential utility of converted GCaMP for targeted imaging, detailed characterization of the red GCaMP state and the photoconversion mechanism might be of basic photophysical interest as well.

In summary, the discovery of the green-to-red photoconversion of GCaMPs presents novel use cases for these established reagents. Red forms of existing GCaMPs have a relatively low sensitivity to calcium. Improvement of the calcium sensitivity of the photoconverted red form through targeted screenings [9, 10] will increase applicability.

## Materials and Methods

### Fly strains

Fruit flies, *Drosophila melanogaster*, were maintained on standard cornmeal food at room temperature. GCaMP transgenic flies, UAS-mCD8GFP, *IR8a*-GAL4 [17] and GR38H02-GAL4 [38] flies are previously described.

### DNA constructs

GCaMP constructs based on pCMV mammalian expression vector were purchased from Addgene Inc. Point mutations were introduced using Quickchange mutagenesis (Life Technologies) following standard protocols. All constructs were sequenced to confirm the GCaMP coding sequence.

### Calcium imaging

Calcium imaging experiments were performed using a two-photon microscope as previously described [16]. The dissected fly brains were pinned down on a silicone plate. A custom-built perfusion system was used to exchange the solution covering the brain. The control solution contains 108mM NaCl, 2mM CaCl_2_, 8.2mM MgCl_2_, 4mM NaHCO_3_, 1mM NaH_2_PO_4_, 5mM trehalose, 10mM sucrose, 5mM HEPES (pH7.4). The depolarizing buffer contains 40mM KCl. Calcium response were generally observed ~2 minutes after exposure of the brain to the depolarizing solution.

### Photoconversion of GCaMPs

Photoconversion was carried out using brains from ~2–5 day old transgenic flies expressing *IR8a*-GAL4; UAS-GCaMP3 or flies carrying *NP225*-GAL4; UAS-GCaMP6s (Figs 1 and 2) and flies expressing GR38H02-GAL4; UAS-mCD8GFP (S3 Fig). Fly brains were dissected in phosphate buffered solution (1xPBS) at room temperature. Dissected brains were mounted onto glass slides and covered with coverslips. Photoconversion was then performed by exposing the brains to light from a mercury lamp (HBO100, Zeiss) through a FITC filter on a Zeiss microscope and a 63x oil-immersion lens with high numerical aperture (NA). Other objectives such as 10x and 20x lens with low NA can be used to achieve photoconversion, but require longer exposure to light. Green and red fluorescent micrographs were taken using a confocal microscope before and after photoconversion. GCaMP photoconversion was also achieved using a Xenon light source with adjustable wavelength (Polychrome V, Till photonics).

For GCaMP photoconversion in mammalian cells, HEK293 cells were cultured on coverslips and transfected with GCaMP constructs using Lipofectamine 2000 reagent (Life Technologies). Two days after transfection, coverslips containing GCaMP-expressing cells were dipped into 1xPBS for 10 seconds and then mounted onto glass slides. Photoconversion was subsequently performed following the same procedure as in fly brains.

For oxidative and anaerobic photoconversion, HEK293 cells were cultured and transfected in glass bottom 12-well culture dishes (MatTek Corporation). Two days after transfection, photoconversion experiments were performed. For anaerobic photocoversion, the culture medium was replaced with serum-free phenol red-free DMEM in the presence of oxygen depleting reagents (30μg/ml catalase, 4.5mg/ml glucose and 250μg/ml glucose oxidase) for 30 minutes in a 37°C incubator immediately before photoconversion. For oxidative photoconversion, the culture medium was replaced with serum-free phenol red-free DMEM in the presence of an electron acceptor (5mM potassium ferricyanide or 5mM benzoquinone) for 30 minutes in 37°C. The treated cells in the 12-well dish were then exposed to blue light on an inverted Olympus microscope with a 40x objective lens. Conversion of purified GCaMP3 was performed in the presence or absence of 5mM potassium ferricyanide and exposed to blue light for a minimum for 2mins. Conversion was performed on an inverted Olympus microscope with a 20x objective lens.

### Determining the red-to-green ratio

Dissected fly brain expressing *IR8a*-GAL4 and UAS-GCaMP3 were mounted to glass slides with 1xPBS used as mounting buffer. Photoconversion was performed as described above. To determine the red-to-green ratio, the strongest cluster of glomerular signals was selected and the mean green and red fluorescence at different time points (green signal: 0sec is set at 100%; red signal: 120sec is set at 100%) were calculated using Image J software.

## Supporting Information

S1 Fig GCaMP3 expressed in central brain neurons can be converted from green to red fluorescence. Fluorescent micrographs of a dissected *Drosophila* brain expressing UAS-GCaMP3 under the control of *NP225*-GAL4, which drives expression in projection neurons and other central neurons in fly brain. The brain was exposed to a blue light source (mercury arc light passed through a Zeiss 60x oil-immersion objective) for different durations as indicated on top of each panel. Green (top) and red (bottom).
(TIF)

S2 Fig Photo-conversion of GCaMP variants. **(A)** Different GCaMP proteins were expressed in HEK293 cells. Green (top), red (middle) and merged (bottom) fluorescent micrographs were taken after the cells were exposed to blue light (mercury arc light passed through a Zeiss 60x oil-immersion objective) for 5min.**(B)** A fly brain expressing *IR64a*-GAL4; UAS-GCaMP1.6 was exposed to blue light for different amounts of time as indicated above the panel. Green (top) and red (bottom) fluorescent confocal micrographs were taken. Note that GCaMP1.6 did not convert photoconvert; rather it was bleached.
(TIF)

S3 Fig GFP can be photo-converted from green to red under similar conditions. Brain from fly expressing GR38-GAL4; UAS-mCD8GFP were dissected and subjected to blue light induced photo-conversion. Top: green channel; middle: red channel; bottom: merged.
(TIF)

S4 Fig Converted red fluorescence of purified GCaMP decays after several hours. Purified GCaMP3 was subjected to blue light-induced photoconversion in the presence of 5mM potassium ferricyanide and monitored for several hours. Top: green channel; bottom: red channel.
(TIF)
