# Supplementary figures and images for: Green-to-Red Photoconversion of GCaMP
Source: PLoS One. 2015 Sep 18;10(9):e0138127. doi: 10.1371/journal.pone.0138127 (PMC4575167; doi:10.1371/journal.pone.0138127)

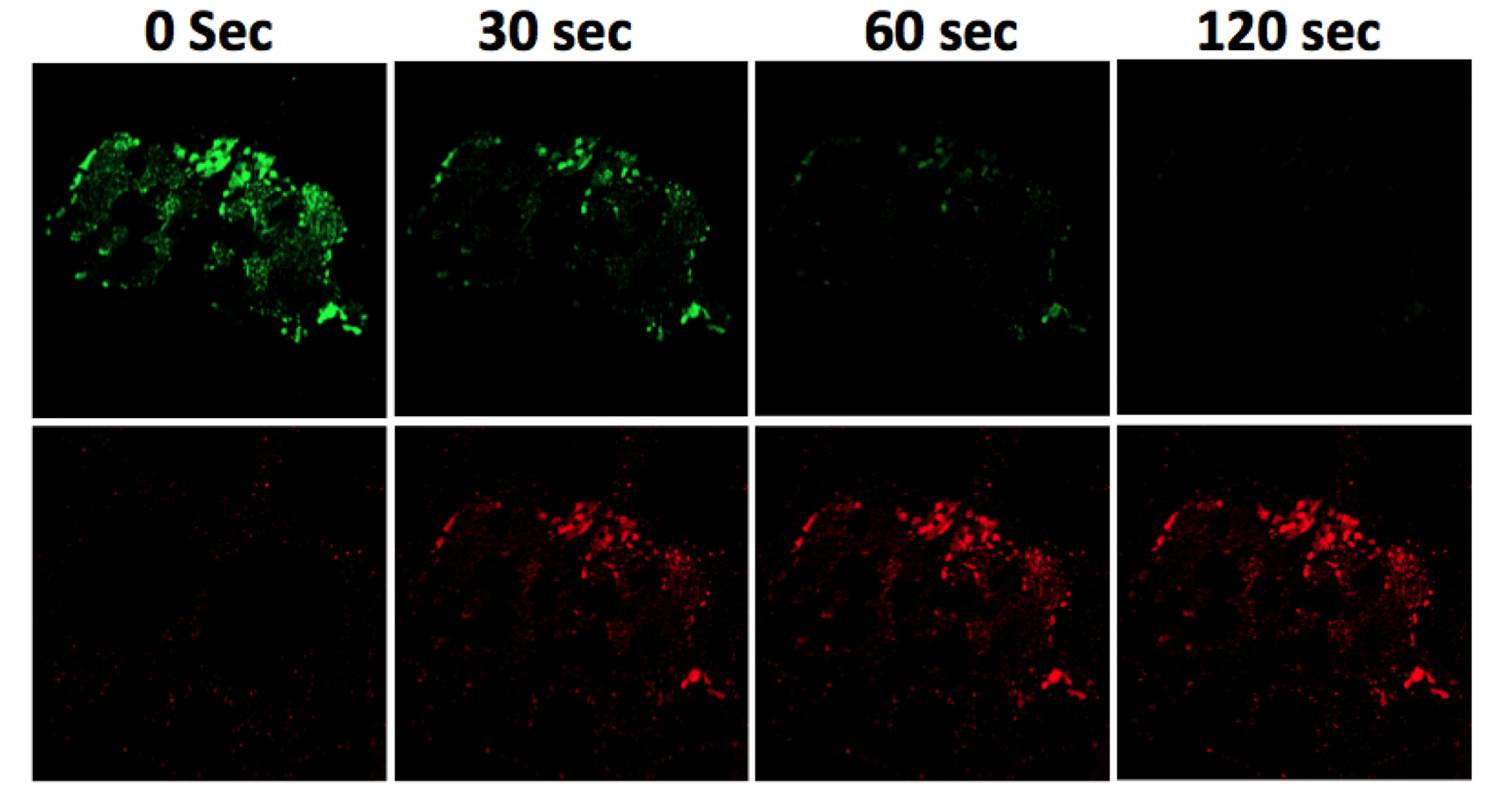

Supplement: S1 Fig — Fluorescent micrographs of a dissected Drosophila brain expressing UAS-GCaMP3 under the control of NP225-GAL4, which drives expression in projection neurons and other central neurons in fly brain. The brain was exposed to a blue light source (mercury arc light passed through a Zeiss 60x oil-immersion objective) for different durations as indicated on top of each panel. Green (top) and red (bottom). (TIF) [file pone.0138127.s001.tif]

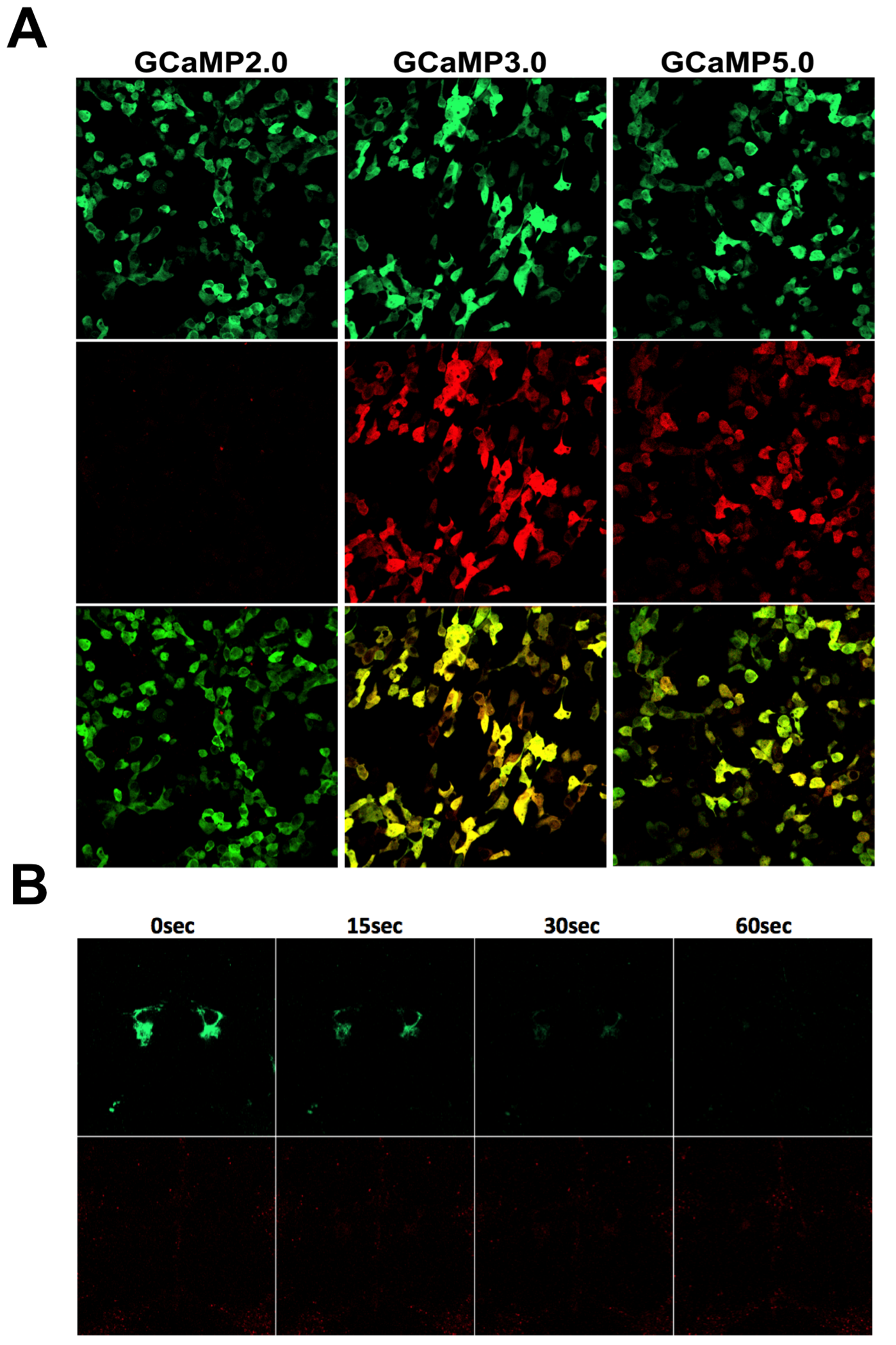

Supplement: S2 Fig — (A) Different GCaMP proteins were expressed in HEK293 cells. Green (top), red (middle) and merged (bottom) fluorescent micrographs were taken after the cells were exposed to blue light (mercury arc light passed through a Zeiss 60x oil-immersion objective) for 5min.(B) A fly brain expressing IR64a-GAL4; UAS-GCaMP1.6 was exposed to blue light for different amounts of time as indicated above the panel. Green (top) and red (bottom) fluorescent confocal micrographs were taken. Note that GCaMP1.6 did not convert photoconvert; rather it was bleached. (TIF) [file pone.0138127.s002.tif]

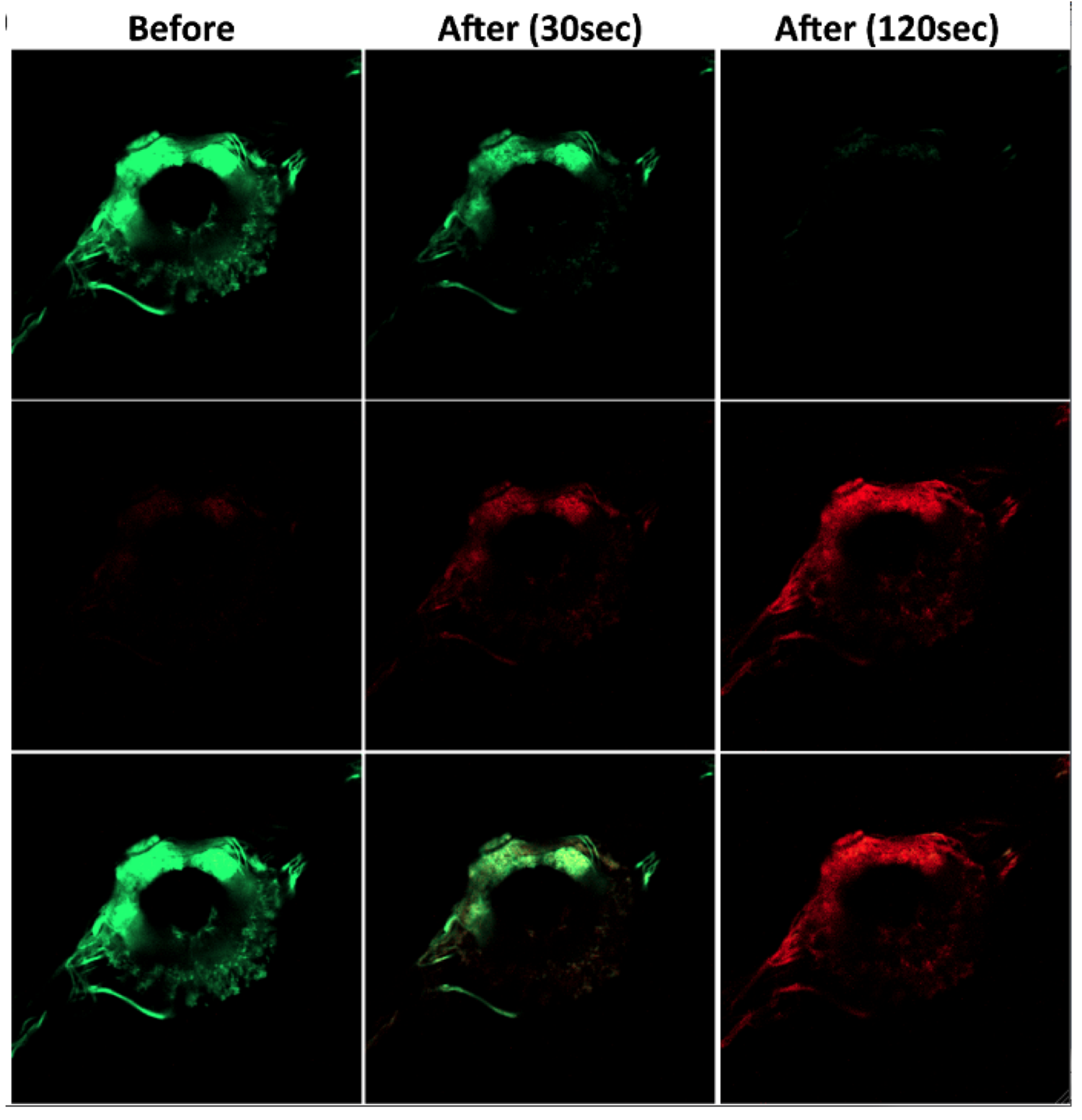

Supplement: S3 Fig — Brain from fly expressing GR38-GAL4; UAS-mCD8GFP were dissected and subjected to blue light induced photo-conversion. Top: green channel; middle: red channel; bottom: merged. (TIF) [file pone.0138127.s003.tif]

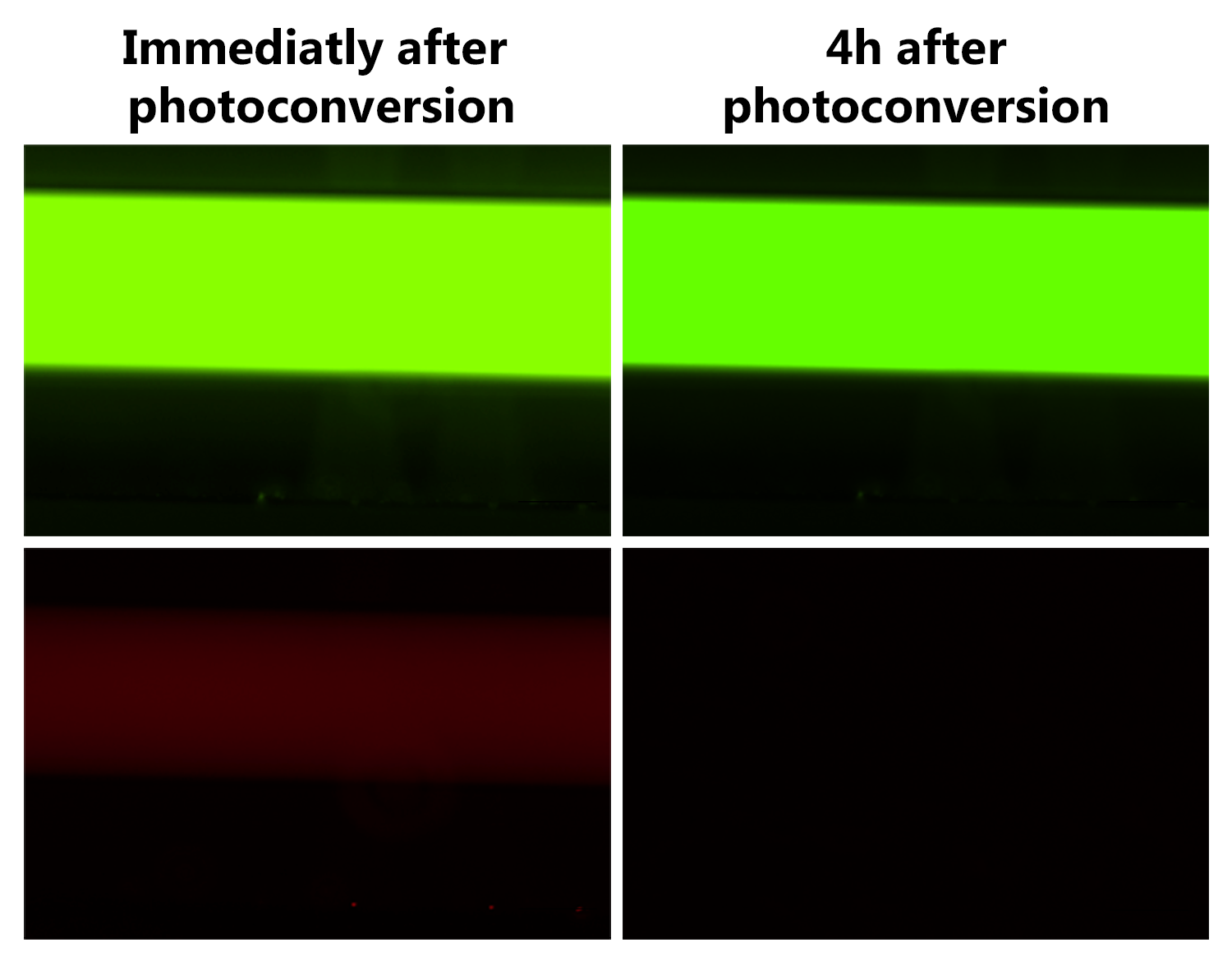

Supplement: S4 Fig — Purified GCaMP3 was subjected to blue light-induced photoconversion in the presence of 5mM potassium ferricyanide and monitored for several hours. Top: green channel; bottom: red channel. (TIF) [file pone.0138127.s004.tif]
